# The impact of regressed endometrial hyperplasia on reproductive outcomes following frozen embryo transfer: a propensity score-matched cohort study

**DOI:** 10.3389/fendo.2026.1846830

**Published:** 2026-06-08

**Authors:** Qinling Zhu, Lizhen Xu, Bing Xu, Yao Lu, Zhe Wei, Wenchao Zhang, Yiwen Meng, Chongwen Shao, Mengjia Shi, Yaqiong He, Jiangan Huang, Yuan Wang, Jia Qi, Ying Ding, Yun Sun

**Affiliations:** Department of Reproductive Medicine, Shanghai Key Laboratory for Assisted Reproduction and Reproductive Genetics, Ren Ji Hospital, Shanghai Jiao Tong University School of Medicine, Shanghai, China

**Keywords:** conservative treatment, endometrial hyperplasia, frozen embryo transfer, live birth, propensity score matching

## Abstract

**Purpose:**

To investigate the impact of regressed endometrial hyperplasia (EH) on assisted reproductive technology outcomes, particularly in comparison with infertile women with a relatively normal endometrium.

**Methods:**

This retrospective study included 95 patients with EH regression after conservative treatment and 30634 infertile controls at the Reproductive Medicine Center from January 2015 to December 2022. Propensity score matching was performed at a ratio of 1:4 between EH and control patients following first frozen embryo transfer. Pregnancy, obstetric and neonatal outcomes were compared between matched pairs. Subgroup analysis was performed between EH patients with and without atypia. Binary logistic regression was conducted to identify risk factors for live birth among EH patients. The primary outcome was live birth rate.

**Results:**

Compared with matched controls, patients with EH exhibited a significantly lower live birth rate (31.6% vs. 49.2%, *P* = 0.002), reduced clinical pregnancy rate (44.2% vs. 59.2%, *P* = 0.008), and higher pregnancy loss rate (40% vs. 26.1%, *P* = 0.046). Subgroup analysis demonstrated a marginally lower live birth rate in EH patients with atypia compared with those without atypia. Furthermore, logistic regression analysis confirmed that EH with atypia, prolonged progesterone treatment and delayed remission associated with reduced likelihood of live birth.

**Conclusions:**

Even after complete remission, EH remained associated with a reduced likelihood of live birth following FET. Prolonged progesterone therapy and delayed remission further diminished the live birth likelihood, highlighting the need for optimized treatment and individualized conception counseling in this high-risk population.

## Introduction

Endometrial hyperplasia (EH) encompasses a spectrum of irregular morphological changes characterized by abnormal proliferation of endometrial glands during the proliferative phase of menstrual cycle (1). According to the latest World Health Organization criteria, EH is classified into EH with and without atypia based on the presence or absence of cellular atypia (2). The frequency of EH is approximately 2‰ among reproductive-aged women (3), but is 5–10 times higher in those with infertility (4), highlighting the importance of fertility management in this population.

High-dose oral progestins, a levonorgestrel-releasing intrauterine system or combined treatments have demonstrated effectiveness in treating EH, with regression rate ranging from 70% to 90% (5–7). These options serve as alternatives for women who strongly desire fertility preservation. Women with EH are at a higher risk of infertility due to increased occurrence of anovulation, obesity and metabolic disorders. Therefore, assisted reproductive technology (ART) is commonly recommended to improve the chances of pregnancy and reduce the interval to conception (8).

Although ART has been shown to be effective in achieving successful pregnancies in EH patients across numerous studies (9–13), a recent retrospective study first reported that, compared with infertile women with normal endometrium, 42 women with atypical EH exhibited a marginally lower live birth rate after fresh embryo transfer (14). This suboptimal outcome may be attributed to impaired endometrial receptivity possibly due to abnormal estradiol and progesterone signaling (15). In fresh embryo transfer, supraphysiological estradiol levels may further compromise endometrial receptivity (16), particularly in individuals with EH who may be more sensitive to fluctuations in estradiol. Whereas in frozen embryo transfer (FET), the endometrium is exposed to estradiol levels that are closer to physiological levels, which may be favored for individuals with EH (17). Notably, no prior studies have investigated the impact of EH on reproductive outcomes in FET compared to normal patients. Furthermore, whether the progesterone treatment or the persistence of EH prior to remission affects reproductive outcomes remains unknown.

In this study, we aimed to investigate reproductive outcomes, as well as maternal and neonatal complications, in a cohort of 95 EH patients who achieved complete remission following conservative treatment and 30,634 control patients with a relative normal endometrium undergoing their first FET. A subgroup analysis was also performed on EH patients with and without atypia to investigate the impact of disease severity on reproductive outcomes.

## Materials and methods

### Study population and design

This was a retrospective cohort study, and data were collected from the Center of Reproductive Medicine, Renji Hospital, Shanghai Jiaotong University School of Medicine between January 2015 and December 2022 from patients who undergoing *in vitro* fertilization or intracytoplasmic sperm injection (IVF/ICSI) treatment. Reproductive outcomes, maternal and neonatal complications were compared between women with and without EH undergoing their first FET. This study was approved by the Ethics Committee of Renji Hospital, Shanghai Jiaotong University School of Medicine (approval number 2015030308).

The inclusion criteria for patients with EH were as follows: (1) histopathologically confirmed diagnosis of EH by two gynecological pathologists independently; (2) completion of standard conservative treatment with regression prior to FET; and (3) adherence to standard controlled ovarian stimulation protocols and IVF/ICSI treatment. The exclusion criteria were: (1) cases involving preimplantation genetic testing cycles; (2) cases without embryo transfer; (3) reproductive system malformations, including septate uterus, unicornuate uterus, and other conditions; (4) endometriosis or adenomyosis; and (5) early-stage endometrial cancer. Subsequently, 30,634 infertile women who showed no histopathological evidence of EH following endometrial biopsy, were included in the control group after screening for the exclusion criteria.

### Conservative treatment for patients with EH

All EH patients received progestin therapy. Oral megestrol acetate was administered at 40–80 mg/day for EH without atypia, and 160–320 mg/day for EH with atypia, until two consecutive biopsies confirmed the histological regression. During treatment, endometrial evaluation was performed every 3 months by hysteroscopy and biopsy to assess therapeutic response and guide subsequent management.

### IVF/ICSI-FET treatment

Controlled ovarian stimulation with GnRH-antagonist was performed in the enrolled patients under a routine procedure in our center. When two or more follicles reached a diameter greater than 17 mm, recombinant human chorionic gonadotropin (hCG, 250 μg) was administered to trigger oocyte maturation. Oocytes were then fertilized via either conventional IVF or ICSI. All patients enrolled in this study adopted a whole embryo frozen strategy. Endometrial preparation and FET were performed in natural, stimulated or hormone replacement cycles after endometrial lesions remission. One or two thawed-cleavage high-quality embryos were transferred on day 3 after initiating luteal phase support, while one high quality thawed-blastocyst was scheduled for transfer on day 5. According to Puissant criteria, good-quality cleavage embryos were those demonstrating 7–10 cells with ≤ 20% fragmentation. For blastocyst embryos, a morphology scoring of ≥ 4BC based on Gardner criteria were considered good-quality embryo. Regular luteal phase support was initiated with vaginal progesterone gel and oral dydrogesterone once the endometrium reached optimal thickness, and continued until 10 to12 weeks of gestation.

### Outcomes measurement

The primary outcome was the live birth rate, defined as delivery of a live neonate beyond 28 weeks. Secondary outcomes included biochemical pregnancy rate, clinical pregnancy rate, and pregnancy loss rate. Biochemical pregnancy was defined as the detection of serum β-hCG ≥10 IU/L measured 12 to 14 days after embryo transfer. Clinical pregnancy was confirmed by the observation of an intrauterine gestational sac via transvaginal ultrasound around 35 days after embryo transfer. Pregnancy loss was defined as pregnancy resulting in a spontaneous abortion before 28 weeks of gestation, including biochemical pregnancy loss. For maternal and neonatal complications, we assessed the rate of gestational hypertension, gestational diabetes mellitus (GDM), preterm birth (delivery between 28 and 37 weeks of gestation), cervical insufficiency (cervical length less than 25 mm along with progressive cervical dilation and shortening before 24 weeks of gestation), macrosomia (birth weight > 4000 g), and low birthweight (birth weight < 2500 g beyond 28 gestational weeks). Birth defects were evaluated according to the International Classification of Diseases, 10^th^ edition (ICD-10).

### Statistical analysis

Data were analyzed using IBM SPSS Statistics (SPSS Inc, version 21, IBM Corp., Armonk, NY, USA). Continuous variables were presented as mean ± SD, and comparisons were made using Student’s *t-* test for normally distributed data. For non-normally distributed data, results were presented as medians (Quartile 1– Quartile 3) and compared using the Mann–Whitney *U* test. Categorical variables were presented as percentages (%) and frequencies (n) and compared using Chi-squared test, with the Fisher exact test for expected frequencies less than 5. A two-sided *P*-value < 0.05 was considered statistically significant for all comparisons.

**Figure 1:**
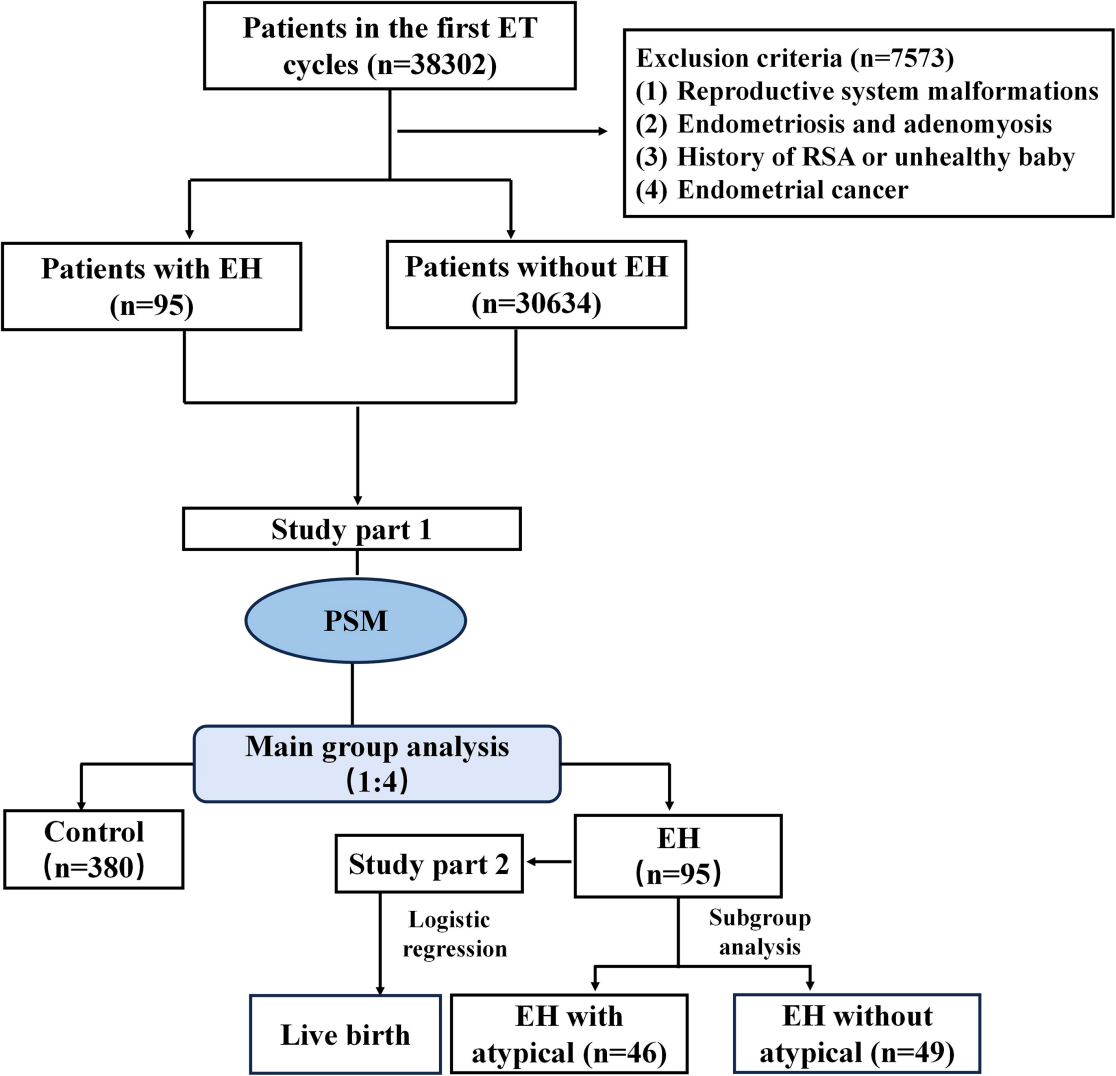
Flow chart of the study on the impact of EH on reproductive outcomes following IVF/ICSI-frozen embryo transfer. IVF, *in vitro* fertilization; ICSI, intracytoplasmic sperm injection; EH, endometrial hyperplasia; ET, embryo transfer; PSM, propensity score match.

In part 1 of the study (**Figure 1**), variables such as age, body mass index (BMI), basal hormone profiles (follicle stimulating hormone (FSH), testosterone (T), thyroid stimulating hormone (TSH)), prevalence of primary infertility, infertility duration, indications for IVF/ICSI, and the stage and number of embryos transferred were matched by propensity score matching (PSM) using the “Match It” package in R software (version 4.0.2, R Foundation for Statistical Computing, Vienna, Austria) to minimize potential confounding factors. Patients with EH were matched to controls at a 1:4 ratio. A subgroup analysis was also conducted between EH patients with and without atypia.

In part 2 of the study (**Figure 1**), binary logistic regression analysis was performed among patients with EH to evaluate potential risk factors for live birth, including EH subtype, the duration of progesterone treatment, duration of EH persistence, number of biopsies, and the interval from remission to FET. Covariates included age, BMI, infertility duration and type, endometrial thickness, and the number and stage of embryos transferred. Both unadjusted and adjusted odds ratios (ORs) and 95% confidence intervals (CIs) were calculated by regression analysis.

## Results

### Baseline characteristics

A total of 95 patients with EH and 30,634 controls without evidence of endometrial abnormalities were enrolled in this study. Among patients with EH, 41 were diagnosed and achieved complete remission before IVF/ICSI treatment, while 54 were diagnosed in the subsequent menstrual cycle after IVF/ICSI treatment and achieved complete resolution before FET. Prior to PSM, the average age, BMI, and duration of infertility in patients with EH were significantly higher than those in the control patients. Moreover, the proportion of patients with primary infertility and those adopting IVF treatment was notably higher in the EH group. Additionally, the indications for IVF/ICSI treatment were different between the two groups (*P* < 0.001). Tubal and anovulatory factors were the primary indications in the EH group, while tubal and male factors were more prevalent in the control group. There was no difference in terms of basal endocrine profiles and TSH levels between the two groups. After PSM, 95 patients with EH were successfully matched to 380 control patients. Following the matching process, the baseline characteristics, including age, BMI, duration of infertility, and indications for IVF/ICSI, were comparable between the two groups. Standardized mean differences (SMDs) < 0.1 indicated a good balance across all covariates (**Table 1**).

**Table 1:** Baseline characteristics of EH and control patients before and after PSM.

Characteristics	EH group (n=95)	Unmatched control (n=30634)	*P* value	Matched control(n=380)	*P* value	*SMD*
Age (yr)	32 (29-36)	30 (27-33)	<0.001	33.0 (30-36)	0.34	0.04
BMI (kg/m^2^)	22.5 (21-25.4)	21.4 (19.7-23.6)	< 0.001	23.0 (20.9-26)	0.49	0.07
Primary infertility, % (n)	76.8 (73/95)	64.1 (19645/30634)	0.01	75.5 (287/380)	0.79	0.01
Parity (%)						
Infertility duration (years)	3.0 (2-5)	2.0 (2-3)	<0.001	3.0 (2-6)	0.22	0.05
Basal FSH (IU/L)	6.7 (5.4-8.1)	6.6 (5.6-7.8)	0.92	6.7 (5.6-7.9)	0.71	0.02
Basal T (nM/L)	0.7 (0.7-1.1)	0.8 (0.7-1.1)	0.94	0.75 (0.7-1.1)	0.67	0.09
TSH (IU/L)	2.1 (1.4-2.7)	2 (1.0-2.6)	0.24	2.1 (1.5-2.8)	0.82	0.02
Indications for IVF/ICSI			< 0.001		0.76	
Tubal factors, % (n)	53.7 (51/95)	36.4 (11166/30634)		55 (209/380)		0.02
Ovulatory dysfunction, % (n)	30.5 (29/95)	19.7 (6025/30634)		29.2 (111/380)		0.02
Male factors, % (n)	10.5 (10/95)	9.4 (2879/30634)		8.2 (31/380)		0.06
Others, % (n)	5.3 (5/95)	34.5(10564/30634)		7.6 (29/380)		0.02
Percentage of IVF, % (n)	56.8 (54/95)	46.4 (14213/30634)	0.04	55.0 (209/380)	0.49	0.03

Data are presented as either medians (Quartile 1– Quartile 3) or % (n). EH, endometrial hyperplasia; BMI, body mass index; FSH, follicle stimulating hormone; T, testosterone; TSH, thyroid stimulating hormone; IVF, *in vitro* fertilization; ICSI, intracytoplasmic sperm injection. SMD, standardized mean differences.

### Outcomes of endometrial preparation and pregnancy

Endometrial preparation for FET was performed in natural, hormone therapy, and stimulated cycles. The distribution of these protocols was similar in the two groups. There was no significant difference in endometrial thickness between the two groups. The number and developmental stage of the transferred embryos were similar in the two groups (**Table 2**).

**Table 2:** Outcomes of endometrial preparation and pregnancy of EH patients and their matched controls.

Characteristics	EH group (n=95)	Matched (n=380)	*Relative ratio* *(95% CI)*	*P* value
Endometrial preparation			NA	0.37
Natural cycles, % (n)	8.4 (8/95)	8.7 (33/380)		
Hormone therapy cycles, % (n)	73.7 (70/95)	78.9 (300/380)		
Stimulated cycles, % (n)	17.9 (17/95)	12.4 (47/380)		
Endometrium thickness (mm)	8.8 (7.8-10)	8.7 (7.5-10)		0.33
No. of embryos transferred (n)	1.0 (1-2)	1.0 (1-2)	NA	0.88
Stage of embryo transfer, % (n)			NA	0.74
D3	37.9 (36/95)	39.7 (151/380)		
D5/D6	62.1 (59/95)	60.3 (229/380)		
Biochemical pregnancy rate, % (n)	52.6 (50/95)	66.6 (253/380)	0.79 (0.65-0.97)	0.01
Clinical pregnancy rate, % (n)	44.2 (42/95)	59.2 (225/380)	0.75 (0.59-0.95)	0.01
Pregnancy loss rate, % (n)	40.0 (20/50)	26.1 (66/253)	1.53 (1.03-2.28)	0.046
Biochemical	16.0 (8/50)	11.1 (28/253)	1.45 (0.7-2.98)	0.33
First trimester	18.0 (9/50)	13.4 (34/253)	1.34 (0.69-2.6)	0.40
Second trimester	6.0 (3/50)	1.6 (4/253)	3.80 (0.88-16.4)	0.09
Live birth rate, % (n)	31.6 (30/95)	49.2 (187/380)	0.64 (0.47-0.88)	0.002
Singleton	29.5 (28/95)	46.3 (176/380)		
Twin	2.1 (2/95)	2.9 (11/380)		

Data are presented as either medians (Quartile 1– Quartile 3) or % (n). EH, endometrial hyperplasia.

The live birth rate in the unmatched control cohort was 52% (15, 937/30, 634). After PSM, the live birth rate was 49.2%. Compared with matched controls, patients with EH had a significantly lower live birth rate (31.6% vs. 49.2%, *P* = 0.002), as well as reduced biochemical (52.6% vs. 66.6%, *P* = 0.01) and clinical pregnancy rate (44.2% vs. 59.2%, *P* = 0.01) (**Table 2**). The overall pregnancy loss rate was higher in individuals with EH. However, no significant differences were found in biochemical, first trimester, and second trimester pregnancy loss between the two groups (**Table 2**).

### Maternal and neonatal outcomes

The incidence of cervical insufficiency was higher in the EH group compared with matched controls (4.8% vs. 0%, *P* = 0.024) (**Table 3**). The rate of gestational hypertension, gestational diabetes mellitus, and preterm delivery were comparable between the two groups (**Table 3**). Similarly, no significant differences were observed in neonatal outcomes, including macrosomia and low birthweight. No birth defects were observed in either EH group or control group.

**Table 3:** Maternal and neonatal complications between EH patients and matched groups.

	EH group (n=95)	Matched (n=380)	*P* value
Maternal			
Gestational hypertension, % (n)	4.8 (2/42)	8 (18/225)	0.46
Gestational diabetes mellitus, % (n)	4.8 (2/42)	11.1 (25/225)	0.21
Preterm delivery rate, % (n)	11.9 (5/42)	12 (27/225)	0.99
Cervical insufficiency, % (n)	4.8 (2/42)	0	0.024
Neonatal			
Birthweight (g)	3360.8 ± 552.4	3273.7 ± 593.8	0.45
Low birthweight, % (n)	6.3 (2/32)	8.1 (16/198)	1.00
Macrosomia, % (n)	12.5 (4/32)	7.1 (14/198)	0.29

Data are presented as either medians (Quartile 1– Quartile 3) or mean ± SD or % (n). EH, endometrial hyperplasia.

### Subgroup analysis of endometrial hyperplasia with and without atypia

Patients with EH were subdivided into two groups based on the presence or absence of cytological atypia. Among these patients, 46 had atypical features, whereas 49 had non-atypical characteristics. The proportion of primary infertility was higher in EH patients with atypia, while other baseline characteristics were comparable between two groups (**Supplementary Table 1**). The number and developmental stage of transferred embryos, endometrial thickness, and endometrial preparation protocols did not differ between the two subgroups (**Table 4**). However, EH patients with atypia had a longer duration of progesterone treatment, prolonged persistence of EH, a greater number of endometrial biopsies, and a longer interval from remission to FET. Notably, the live birth rate was marginally lower in EH patients with atypia (21.7% vs. 40.8%, *P* = 0.046), while the clinical pregnancy rate and pregnancy loss rate were comparable between the two groups (**Table 4**).

**Table 4:** Outcomes of endometrial preparation and pregnancy of EH patients with and without atypia.

	EH with atypia (n=46)	EH without atypia (n=49)	*P* value
Progesterone treatment duration (months)	6 (4.3-10)	3 (3-4)	<0.001
EH persistence duration (months)	7 (4.25-9)	2.5 (2-4)	<0.001
Number of biopsies (n)	3 (2-3.5)	2 (2-2)	<0.001
Time from remission to FET (months)	2.5 (1-7)	1.5 (1-3.25)	<0.001
Endometrial preparation			0.14
Natural cycles, % (n)	13 (6/46)	4.1 (2/49)	
Hormone therapy cycles, % (n)	67.4 (31/46)	79.6 (39/49)	
Stimulated cycles, % (n)	19.6 (9/46)	16.3 (8/49)	
Endometrium thickness (mm)	8 (6.7-10)	8.7 (7.2-9.7)	0.19
No. of embryos transferred (n)	1 (1-2)	1 (1-2)	0.51
Stage of embryo transfer, % (n)			0.81
D3	39.1 (18/46)	36.7 (18/49)	
D5/D6	60.9 (28/46)	63.3 (31/49)	
Biochemistry pregnancy, % (n)	45.7 (21/46)	59.2 (29/49)	0.87
Clinical pregnancy rate, % (n)	39.1 (18/46)	49 (24/49)	0.33
Pregnancy loss rate, % (n)	52.4 (11/21)	31 (9/29)	0.13
Biochemical	14.3 (3/21)	17.2 (5/29)	0.44
First trimester	28.6 (6/21)	10.3 (3/29)	0.098
Second trimester	9.5 (2/21)	3.4 (1/29)	0.57
Live birth rate, % (n)	21.7 (10/46)	40.8 (20/49)	0.046
Singleton	19.6 (9/46)	38.8 (19/49)	
Twin	2.1 (1/46)	2.0 (1/49)	

Data are presented as either medians (Quartile 1– Quartile 3) or %.

### Binary logistic regression analysis of risk factors for live birth in EH patients

Unadjusted and adjusted ORs and 95% CIs of the potential risk factors associated with live birth in EH patients are shown in **Table 5**. Consistent with subgroup analysis findings, EH patients with atypia had lower odds of live birth both before and after adjustment (crude OR,0.40[95% CI, 0.16-0.99]; *P* < 0.048; adjusted OR,0.34[95% CI, 0.13-0.92]; *P* = 0.034). Moreover, longer progesterone treatment duration (adjusted OR,0.83[95% CI, 0.71–0.97]; *P* = 0.021) and extended EH persistence before remission (adjusted OR 0.79, [95% CI, 0.65–0.96]; *P* = 0.018) were identified as independent risk factors for a reduced likelihood of live birth, whereas the interval from EH remission to FET and the number of biopsies showed no significant association with live birth.

**Table 5:** Crude and adjusted odds ratios of the risk factors for live birth in EH patients.

	Live birth rate			
Variables	Crude OR (95% CI)	*P* value	Adjusted OR (95% CI)	*P* value
EH phenotype				
Without atypia	Reference		Reference	
With atypia	0.40 (0.16-0.99)	0.048	0.34 (0.13-0.92)	0.034
Progesterone treatment duration (months)	0.86 (0.76-0.98)	0.025	0.84 (0.72-0.98)	0.028
EH persistence duration (months)	0.82 (0.7-0.97)	0.019	0.79 (0.65-0.96)	0.018
No. of biopsies (n)	0.79 (0.53-1.17)	0.24	0.72 (0.44-1.17)	0.18
Time from remission to FET (months)	1.03 (0.96-1.12)	0.42	0.90(0.76-1.06)	0.21

EH, endometrial hyperplasia; BMI, body mass index; OR, odds ratio; CI, confidence interval.

## Discussion

In this study, we provide evidence that women with EH who achieved complete remission after conservative treatment still exhibited significantly lower live birth rate following the first FET cycle, even after adjustment for confounding factors using PSM. These findings suggest that histological remission may not fully restore endometrial function, and that persistent impairment of endometrial receptivity may continue to affect implantation and pregnancy maintenance.

In contrast to natural conception, ART treatment has been shown to significantly increase the likelihood of successful pregnancy in EH patients with atypia and early-stage endometrial cancer (13). However, only one previous study has specially addressed the impact of atypical EH on reproductive outcomes, showing that EH patients with atypia experiencing a higher rate of pregnancy loss and preterm delivery after fresh embryo transfer compared to matched infertile controls, although no significant difference in live birth was observed due to limited sample size (14). Here in this study, we further demonstrated found that patients with EH had a lower live birth rate than matched infertile controls. Importantly, subgroup analyses demonstrated that patients with atypical EH had lower live birth rate compared with those without atypia, suggesting that the severity of endometrial pathology may be closely associated with impaired reproductive outcomes. Together, these findings support the concept that histological remission may not fully restore normal endometrial function, particularly in patients with more severe disease.

Although the precise mechanisms for the poor reproductive outcomes in EH remain unclear, several observations from our study provide potential mechanistic insights. We found that prolonged progesterone treatment and extended EH persistence prior to remission were independently associated with the decreased live birth rate among EH patients, suggesting disease persistence and prolonged hormonal exposure may exert long-term effects on endometrial function. Previous studies have demonstrated that high-dose progestin treatment suppresses progesterone receptor expression, which may disturb the actions of progesterone on endometrium even after regression, thereby impairing endometrial receptivity and decidualization (18, 19). Additionally, HOXA10, a critical marker of endometrial receptivity, has been found to be downregulated in the endometrium of EH patients, potentially compromising endometrial receptivity (20).A recent proteomic study further suggest that abnormal energy metabolism, coagulation pathways, and endocrine signaling may contribute to persistent endometrial dysfunction after fertility-preserving treatment (15). Collectively, these findings indicate that residual molecular and functional abnormalities may persist despite pathological remission of EH. Further research is needed to unravel the intricate molecular mechanisms underlying persistent endometrial dysfunction following fertility-preserving treatment for EH.

Another concern regarding reproductive challenges among EH patients is the limited understanding of maternal and perinatal complications. Song et al. found that after fresh embryo transfer, the preterm birth rate was substantially higher in women with atypical EH than matched controls (14). However, this phenomenon was not observed in our study which may be attributable to the inclusion of both EH patients with and without atypia. Moreover, a higher incidence of cervical insufficiency was observed among EH patients in our study. However, this finding was based on only two cases and should therefore be interpreted with caution. Further studies with larger sample sizes are needed to validate this finding. Other maternal and neonatal complications were similar between EH patients and the controls. These consistency outcomes may result from the effective matching of age, BMI, and infertility indicators using PSM.

Our study’s key strength is the relatively large sample size of EH patients compared to previous studies, which enhances the reliability our findings when comparing reproductive outcomes to control patients. PSM was applied to further minimize confounding factors in evaluating reproductive outcomes between EH and control patients, independent of patients’ baseline characteristics. Moreover, our study comprehensively evaluated reproductive outcomes, maternal and neonatal complications, providing a complete perspective on the impact of EH in patients undergoing FET.

Nonetheless, several limitations should be noted in this study. First, although PSM was employed to adjust for confounding factors, the single-center, retrospective design of the study may introduce potential selection bias. Second, the sample size of EH patients, particularly in subgroup analyses, was relatively limited, potentially reducing statistical power. Third, fasting glucose, insulin, LH, and E2 were not included in the matching model because of incomplete data, and residual confounding cannot be entirely excluded. Finally, mechanistic interpretations regarding progesterone receptor suppression and persistent endometrial dysfunction remain speculative and require further experimental validation.

## Conclusion

Our study demonstrated that even after complete remission, EH was still associated with a reduced live birth rate in FET cycles. Prolonged progesterone therapy and delayed EH remission further decreased the odds of live birth. These findings highlight the need for optimized progestin treatment strategies and individualized fertility counseling in this high-risk population. Larger prospective studies are warranted to validate these results.

## Data Availability

The original contributions presented in the study are included in the article/**Supplementary Material**. Further inquiries can be directed to the corresponding author.
