# Supplementary material for: The impact of regressed endometrial hyperplasia on reproductive outcomes following frozen embryo transfer: a propensity score-matched cohort study
Source: Front Endocrinol (Lausanne). 2026 Jun 8;17:1846830. doi: 10.3389/fendo.2026.1846830 (PMC13283882; doi:10.3389/fendo.2026.1846830)
Supplement: Supplementary file 1 [file Table1.docx]

**Supplemental Table 1. Baseline characteristics between EH with and without atypia**

|  | EH with atypia (n=46) | EH without atypia (n=49) | *P* value |
| --- | --- | --- | --- |
| Age (years) | 30.5 (28-33) | 31 (29-36.7) | 0.256 |
| BMI (kg/m^2^) | 22.1 (20.4-25.4) | 22.3 (21.4-24.3) | 0.941 |
| Primary infertility, % (n) | 87 (40/46) | 67.3 (33/49) | 0.024 |
| Infertility duration (years) | 3 (2-5) | 4 (2.75-5.25) | 0.728 |
| Basal FSH (IU/L） | 6.8 (5.5-8.5) | 6.4 (5.1-7.7) | 0.223 |
| Basal T (nM/L) | 0.9 (0.68-1.31) | 0.7 (0.68-1.04) | 0.225 |
| TSH (IU/L) | 2.1 (1.5-2.8) | 2.3 (1.6-2.8) | 0.677 |
| Indications for IVF/ICSI |  |  | 0.957 |
| Tubal factors, % (n) | 52.2 (24/46) | 55.1 (27/49) |  |
| Ovulatory dysfunction, % (n) | 32.6 (15/46) | 28.6 (14/49) |  |
| Male factors, % (n) | 10.9 (5/46) | 10.2 (5/49) |  |
| Others, % (n) | 4.3 (2/46) | 6.1 (3/49) |  |
| Percentage of IVF, % (n) | 58.7 (27/46) | 55.1 (27/49) | 0.724 |
